# The interplay of job embeddedness, collective efficacy, and work meaningfulness on teacher well-being: a mixed-methods study with digital ethnography in China

**DOI:** 10.3389/fpsyg.2024.1448446

**Published:** 2024-10-30

**Authors:** Yumei Lei

**Affiliations:** Graduate School, Pre-school Education, Sehan University, Yeongam County, Republic of Korea

**Keywords:** teacher well-being, job embeddedness, collective efficacy, work meaningfulness, mixed-methods, digital ethnography

## Abstract

**Introduction:**

This study examines the influence of teacher job embeddedness, collective efficacy, and work meaningfulness on the psychological well-being of Chinese teachers. The focus is on understanding how these constructs contribute to teacher well-being through a mixed-methods approach.

**Methods:**

A two-stage structural equation modeling (SEM) analysis was conducted using quantitative data collected from 406 in-service Chinese teachers. The study also incorporated a qualitative phase involving digital ethnography within online teaching communities to provide deeper insights into teachers’ experiences.

**Results:**

The quantitative analysis revealed significant positive direct effects of job embeddedness, collective efficacy, and work meaningfulness on psychological well-being, with work meaningfulness showing the strongest effect. The mediating role of work meaningfulness partially explained the relationships between job embeddedness, collective efficacy, and teacher well-being. The qualitative findings supported and expanded upon these results, emphasizing the role of a supportive school community and strong administrative leadership.

**Discussion:**

The results suggest that enhancing job embeddedness, collective efficacy, and work meaningfulness can significantly contribute to teachers’ psychological well-being. These findings provide actionable insights for school leaders and policymakers in the Chinese educational system to foster environments that promote teacher well-being.

## Introduction

The teaching profession is inherently demanding, marked by substantial workloads often exceeding 50 h per week (Darling-Hammond et al., 2020). This encompasses not only instructional time but also lesson planning, grading, and administrative duties, leaving limited space for personal well-being. Additionally, the increasing diversity and complexity of student needs, ranging from learning disabilities and mental health concerns to socioeconomic disparities, further amplify the challenges faced by educators (Rowan et al., 2021). Moreover, the dynamic nature of societal expectations and educational reforms necessitates continuous adaptation and professional development, placing additional pressure on teachers (Stronge et al., 2011). This confluence of demands can engender significant emotional and psychological strain, contributing to concerning rates of stress, burnout, and diminished morale within the teaching profession (Carroll et al., 2022; Schaufeli et al., 2009).

Given the well-established correlation between teacher well-being and student success (Hanushek and Rivkin, 2006), understanding and fostering the former is of paramount importance. Teacher well-being, a multidimensional construct encompassing emotional, psychological, and physical health (Hascher and Waber, 2021), is significantly influenced by the quality of the work environment (Yin et al., 2016). Two environmental factors that have garnered increasing attention in recent research are teacher job embeddedness and teacher collective efficacy.

Job embeddedness, as defined by Mitchell et al. (2001), reflects the strength of an individual’s connection to their workplace, encompassing both on-the-job factors (e.g., positive colleague relationships, perceived fit with the organization) and off-the-job factors (e.g., community involvement, family ties). Although a robust body of research indicates a positive association between job embeddedness and favorable outcomes like job satisfaction, stress reduction, and lower turnover intentions (Ampofo et al., 2017; Khairunisa and Muafi, 2022; Marasi et al., 2016; Porter et al., 2019), recent studies suggest a more nuanced relationship. Notably, conflicting demands between work and personal life stemming from off-the-job embeddedness can elevate work-related stress (Hinrichs, 2023), and individual differences in boundary management and coping strategies can moderate the overall impact of job embeddedness on well-being (Ghosh and Gurunathan, 2015).

Teacher collective efficacy, in contrast, centers on the shared belief among educators in their collective capacity to effect positive student learning outcomes (Goddard et al., 2004; Loughland and Ryan, 2022; Tschannen-Moran and Barr, 2004). High collective efficacy is associated with favorable school climates, mutual support among teachers, and a shared sense of responsibility for student success—all factors contributing to improved teacher well-being (Strahannée Brown et al., 2019; Voelkel and Chrispeels, 2017).

Despite extensive research on job embeddedness, collective efficacy, and teacher well-being, a significant gap remains in understanding how these constructs interact within the unique context of Chinese education. Most existing studies have been conducted in Western contexts (Khairunisa and Muafi, 2022; Voelkel and Chrispeels, 2017), limiting our understanding of how these constructs function in non-Western educational systems. Given the cultural values of collectivism and social connectedness that are prevalent in China (Du and Wei, 2015), the dynamics of job embeddedness, collective efficacy, and well-being may differ significantly in this context. This study addresses this research gap by examining the relationships between these constructs within the Chinese educational system.

This study offers several unique contributions to the existing literature on job embeddedness, collective efficacy, and teacher well-being. By focusing on Chinese university teachers, the study extends our understanding of these constructs beyond Western contexts. Additionally, the introduction of work meaningfulness as a potential mediating factor provides a more nuanced understanding of how job embeddedness and collective efficacy impact well-being. Furthermore, the study employs a mixed-methods approach, combining quantitative data with qualitative digital ethnography, to comprehensively explore the lived experiences of Chinese teachers. This approach allows for a deeper understanding of the complex factors influencing teacher well-being within the Chinese educational context.

## Literature review

### Psychological well-being: key components and impacts

Psychological well-being, a cornerstone of positive mental health, extends beyond the absence of illness (Winefield et al., 2012). Encompassing positive emotions, life satisfaction, self-esteem, and a sense of purpose (Ryff, 1989), it plays a critical role in overall quality of life. Research consistently links it to positive outcomes like better physical health, stronger relationships, and even increased longevity (Bożek et al., 2020; Diener and Chan, 2011). Positive emotions, for instance, bolster cardiovascular health, immune function, and stress resilience (Pressman and Cohen, 2005). Individuals with higher well-being are more likely to adopt healthy behaviors and cope effectively with challenges (Huppert and So, 2013). Two prominent perspectives inform our understanding of well-being: hedonic and eudaimonic. The hedonic perspective emphasizes maximizing pleasure and minimizing pain, focusing on subjective well-being and positive emotions (Diener, 1984). In contrast, the eudaimonic perspective, rooted in Aristotle’s philosophy, highlights living a meaningful and purposeful life (Waterman, 1993). This perspective encompasses personal growth, self-realization, and the pursuit of significant goals (Ryff, 2014).

Personality traits, such as optimism and resilience, play a significant role in shaping how individuals respond to life events, ultimately impacting their overall well-being (Carver and Scheier, 2014; Miranda and Cruz, 2020). Additionally, social connections and support are crucial for positive mental health, underscoring the importance of strong interpersonal bonds (Thoits, 2011).

The workplace also significantly influences psychological well-being. Job satisfaction, a key component of well-being itself, contributes to overall life satisfaction and happiness (Foster et al., 2020; Warr, 1999). A sense of autonomy, opportunities for growth, and positive relationships with colleagues and supervisors are all linked to higher levels of workplace well-being (Deci and Ryan, 2008; Morales-Rodríguez et al., 2020). Furthermore, finding meaning and purpose in one’s work, known as work meaningfulness, is associated with greater well-being (Rosso et al., 2010).

While generally positive, psychological well-being is not static. Life events, stressors, and individual differences can cause fluctuations over time (Diener et al., 2018). Recognizing this dynamic nature is essential for developing interventions and strategies to enhance and sustain positive mental health (Houben et al., 2015). Interventions aimed at improving psychological well-being, such as mindfulness-based practices, positive psychology techniques, and resilience training programs, have shown promise in both clinical and organizational settings (Briner and Walshe, 2015; Kersemaekers et al., 2018; Fava and Tomba, 2009; Seligman et al., 2005; Sulosaari et al., 2022).

For teachers, psychological well-being is influenced by a combination of personal characteristics, environmental factors, and professional experiences. Several studies have explored the intricate link between teachers’ psychological well-being and their professional lives (e.g., Wang et al., 2023; Zhang et al., 2023). Ilgan et al. (2015) found that a positive school work environment enhances teacher well-being, while McInerney et al. (2015) highlighted the role of self-beliefs in teacher commitment and well-being. Greenier et al. (2021) examined teachers’ ability to regulate their emotions, finding that emotional regulation skills contribute to well-being and engagement. Shao (2023) further explored this concept, proposing a model where teacher enthusiasm, self-efficacy, and grit all contribute to positive well-being among English language teachers. Wang et al.'s (2023) meta-analysis suggests that teacher well-being is impacted by a complex interplay of environmental factors, personal characteristics, instructional practices, and emotion regulation strategies.

In conclusion, this body of research underscores the multifaceted nature of teacher psychological well-being. It highlights the importance of fostering positive work environments, nurturing self-beliefs, and developing emotional regulation skills. Future research can delve deeper into specific interventions and strategies that promote well-being and optimize the teaching experience. As we continue to explore the complexities of psychological well-being, integrating hedonic and eudaimonic perspectives offers a comprehensive understanding of a flourishing and fulfilling life. Strategies aimed at promoting well-being can contribute not only to individual happiness but also to societal well-being, emphasizing the importance of fostering positive mental health across diverse contexts.

### Influence of job embeddedness on turnover intentions

Moving beyond traditional measures of job satisfaction and organizational commitment, job embeddedness offers a comprehensive framework for understanding employee retention (Lee et al., 2014). This relatively recent concept explores the reasons why employees stay with their organizations, acknowledging a complex web of factors that tether individuals not only to their jobs but also to their work communities and personal lives (Kiazad et al., 2015; Mitchell et al., 2001). Job embeddedness is comprised of two key components: on-the-job embeddedness and off-the-job embeddedness. On-the-job embeddedness focuses on workplace aspects that encourage retention, such as strong coworker relationships, alignment with organizational culture, and the perceived costs of leaving (Ng and Feldman, 2010). Conversely, off-the-job embeddedness considers personal life factors influencing employee decisions to stay (Burrows et al., 2022). Family ties, community involvement, and extracurricular activities can all play a role (Mitchell et al., 2001).

Empirical studies have consistently demonstrated the power of job embeddedness in predicting turnover intentions (Coetzer et al., 2019). Employees with high levels of embeddedness, both on and off the job, are less likely to leave their organizations due to the intricate network of connections they have built and the perceived high sacrifice of departure (Heritage et al., 2016; Holtom et al., 2008). Interestingly, research suggests that job embeddedness can even supersede job satisfaction and organizational commitment in influencing turnover decisions (Jiang et al., 2012). Even if an employee is dissatisfied or less committed, strong embeddedness can still deter resignation (Lee et al., 2014).

These insights hold valuable implications for recruitment and retention strategies. Organizations can foster strong on-the-job connections through team-building activities, mentorship programs, and initiatives that align individual and organizational values (Felps et al., 2009; Marasi et al., 2016). Recognizing the significance of off-the-job embeddedness underscores the value of supporting work-life balance, offering community engagement opportunities, and implementing flexible work arrangements to enhance employee retention (Feldman et al., 2012; Porter et al., 2019; Lee et al., 2014).

Furthermore, job embeddedness highlights the role of the local community in retention strategies. Organizations that encourage community involvement and support employees in building local connections can increase off-the-job embeddedness (Ramesh and Gelfand, 2010). This makes it more challenging for employees to contemplate leaving not just their job but their community as well. The positive influence of job embeddedness extends beyond retention, impacting employee well-being in various ways (Chan et al., 2019). Studies have shown that job embeddedness weakens the negative effects of workplace stressors on turnover intentions (Khairunisa and Muafi, 2022). Employees with strong job embeddedness are less likely to consider leaving despite experiencing challenges. Similarly, research suggests that strong work relationships foster a sense of embeddedness, which in turn enhances employee well-being (Ahmad et al., 2023).

The benefits of job embeddedness for employee well-being go beyond mitigating negative experiences. Ampofo et al. (2017) documented a positive association between job embeddedness and life satisfaction, suggesting that feeling integrated within the workplace contributes to overall well-being. Steindórsdóttir et al. (2020) further explored this concept by demonstrating that supportive work environments and the fulfillment of psychological needs contribute to job embeddedness, ultimately enhancing employee well-being. Finally, Sun et al. (2012) found that psychological resources like optimism and resilience contribute to feeling embedded within the workplace, leading to better performance.

Taken together, this body of research highlights job embeddedness as a crucial factor influencing employee well-being. By fostering a sense of connection, fit, and value within the workplace, organizations can create a positive work environment that contributes to employee well-being beyond just job satisfaction.

### Teacher collective efficacy: building strong educational communities

Teacher collective efficacy, a powerful construct rooted in social cognitive theory, transcends individual beliefs. It captures the shared conviction among a school’s faculty that they can collectively influence student learning outcomes (Donohoo, 2018; Goddard et al., 2004). This collective belief system goes beyond the sum of individual self-efficacy; it reflects a group’s combined perception of its capacity to organize and execute effective instructional strategies (Goddard et al., 2000; Loughland and Nguyen, 2020). Research consistently demonstrates that schools characterized by high teacher collective efficacy are more likely to implement innovative teaching methods, persevere in supporting struggling students, and ultimately achieve greater student success (Goddard et al., 2015; Schwabsky et al., 2020; Tschannen-Moran and Barr, 2004).

The significance of collective efficacy lies in its emphasis on teacher collaboration and shared vision for educational goals (Loughland and Ryan, 2022). Studies have shown that even after accounting for prior student achievement and socio-economic status, schools with high collective efficacy demonstrate stronger student outcomes (Donohoo, 2017; Goddard et al., 2004). This suggests that collective efficacy empowers schools to overcome challenges and propel student learning forward. Several factors contribute to nurturing teacher collective efficacy. These include teacher perceptions of school leadership, the structure of collaboration within the school, and the school’s track record of success in improving student learning (Klassen et al., 2011). Leadership practices that empower teachers, encourage shared decision-making, and cultivate a supportive and collaborative school culture are particularly effective in fostering collective efficacy (Leithwood and Jantzi, 2008). The dynamics of collective efficacy highlight that it emerges not simply from the aggregation of individual beliefs, but rather from the interactions and sense of community within the educational setting (Voelkel and Chrispeels, 2017). The mechanisms through which collective efficacy translates into student achievement include setting ambitious goals, persisting in the face of difficulties, and employing effective and differentiated instruction tailored to diverse student needs (Donohoo et al., 2018).

Collective teacher efficacy, the shared belief among teachers in their collective ability to positively impact student learning and well-being, is a key predictor of school success (Quirk et al., 2009). Schools with high collective efficacy implement inclusive practices and differentiated instruction more effectively, leading to equitable learning outcomes (Lyons et al., 2016). Collective efficacy is also strongly linked to teacher well-being, as studies show that this shared belief fosters a supportive and empowering work environment (Fathi et al., 2020; Strahannée Brown et al., 2019).

Research has highlighted collective efficacy as a mediator between various factors and teacher well-being. Herrera et al. (2022) found that perceived organizational justice positively affects teacher well-being, with collective efficacy reinforcing this relationship. Similarly, Skaalvik and Skaalvik (2019) demonstrated that positive work environments, characterized by adequate resources and a sense of belonging, enhance collective efficacy, which boosts teacher engagement and well-being. Capone et al. (2018) also linked collective efficacy to better classroom relationships and a stronger sense of community, benefiting both teachers and students.

Liang et al. (2022) examined the role of professional learning communities (PLCs) in China, revealing that PLCs indirectly enhance teacher well-being by increasing self-efficacy, which in turn strengthens collective efficacy. This underscores the value of collaborative learning in promoting teacher well-being.

Overall, collective teacher efficacy is a powerful construct that positively influences both student achievement and teacher well-being. By fostering a shared belief in teachers’ collective ability to succeed, collective efficacy can create supportive and empowering work environments, improve teacher engagement, and ultimately contribute to more equitable and effective schools.

### Work meaningfulness and its impact on employee engagement

Work meaningfulness, a concept capturing the perceived significance and purpose individuals attach to their work, has become a central theme in organizational psychology (Allan et al., 2019; Deci and Ryan, 2000). Deeply rooted in intrinsic motivation theory, meaningful work is closely linked to positive outcomes like job satisfaction, enjoyment, engagement, and overall well-being (Hirschi, 2012; Steger et al., 2012; Xiao et al., 2022). Employees who find their work meaningful experience it as significant, purposeful, and valuable (Rosso et al., 2010). The relevance of work meaningfulness is undeniable. Research consistently demonstrates its association with a multitude of benefits, including increased job satisfaction, higher engagement, reduced absenteeism, and lower turnover intentions (Lips-Wiersma and Morris, 2009; Liu et al., 2023; Pratt and Ashforth, 2003). Meaningful work even translates into enhanced performance; employees who feel their work holds significance are more likely to go above and beyond, exhibiting greater creativity and innovation (Michaelson et al., 2014).

The sources of work meaningfulness are diverse and vary across individuals. Alignment between personal values and work activities, a sense of contributing to something larger than oneself, autonomy in job tasks, and recognition for efforts all contribute to perceiving work as meaningful (Bailey and Madden, 2016; De Boeck et al., 2019). Leadership that emphasizes open communication, transparency, and employee development fosters an environment conducive to finding meaning in work (Schnell et al., 2013). Job design also plays a significant role. Jobs that offer opportunities for skill variety, task significance, autonomy, and feedback are more likely to be perceived as meaningful (Hackman and Oldham, 1976; Han et al., 2021). The social environment is equally important; supportive coworker relationships and a culture that values individual contributions significantly shape perceptions of meaningfulness (Dechawatanapaisal, 2022; Dutton et al., 2014).

While the positive aspects of work meaningfulness are well-documented, research also explores potential downsides. Excessive meaningfulness can lead to workaholism or burnout if individuals neglect other aspects of life due to an overemphasis on work (Bunderson and Thompson, 2009; Oelberger, 2019). Finding a balance between work meaningfulness and well-being across life domains is crucial for both employees and organizations.

A growing body of research highlights the importance of work meaningfulness in promoting employee well-being. Studies show that transformational leadership, psychological safety, and empowerment foster a sense of purpose at work, which enhances well-being (Arnold et al., 2007; Hasan and Kashif, 2021). This suggests that meaningful work environments can empower employees and improve their overall well-being. Beyond leadership and workplace factors, individual characteristics also play a role. Görgens-Ekermans and Steyn (2016) found that traits like optimism and self-efficacy enhance the relationship between work meaningfulness and subjective well-being. Similarly, Golparvar and Abedini (2014) and Hooker et al. (2020) noted that spiritual fulfillment and engagement in meaningful activities contribute to higher job satisfaction and psychological well-being.

Crego et al. (2020) added mindfulness as a factor, showing that a mindful approach to work strengthens the link between meaningfulness and well-being. These findings underscore the multifaceted nature of work meaningfulness and its positive impact on psychological well-being. By fostering purpose, significance, and alignment with personal values, workplaces can create more fulfilling and productive environments for employees.

## The purpose of the study

Drawing on the reviewed literature, this study proposes a model that examines the relationships between teacher job embeddedness, teacher collective efficacy, work meaningfulness, and teacher psychological well-being. The study aims to investigate these relationships through both quantitative and qualitative methodologies, providing a comprehensive understanding of the factors influencing teacher well-being.

The first hypothesis (H1) posits that teacher job embeddedness is directly related to teachers’ psychological well-being. Research suggests a strong positive association between job embeddedness and employee well-being (Feldman et al., 2012; Kiazad et al., 2015; Marasi et al., 2016). When teachers feel embedded within their workplace through strong on-the-job connections, alignment with values, and a sense of community (Mitchell et al., 2001), they are less likely to experience stress or burnout (Hinrichs, 2023; Jiang et al., 2012; Khairunisa and Muafi, 2022). Additionally, off-the-job embeddedness factors like family ties and community involvement can contribute to overall life satisfaction and well-being beyond just work (Ampofo et al., 2017).

The second hypothesis (H2) suggests that teacher collective efficacy is directly related to teachers’ psychological well-being. A growing body of research highlights the positive influence of teacher collective efficacy on teacher well-being (Donohoo, 2017; Strahannée Brown et al., 2019). Schools characterized by high collective efficacy foster supportive and positive climates (Donohoo, 2018; Fathi et al., 2020) where teachers feel a sense of shared belief in their ability to impact student learning (Goddard et al., 2015). This, in turn, contributes to increased feelings of competence, purpose, and ultimately, greater well-being (Strahannée Brown et al., 2019).

The third hypothesis (H3) proposes that work meaningfulness mediates the relationship between teacher job embeddedness and psychological well-being. Job embeddedness can foster work meaningfulness in several ways. Strong on-the-job connections can provide opportunities for collaboration and knowledge sharing, which can contribute to a sense of purpose and the value of one’s work (Bailey and Madden, 2016; Han et al., 2021). Additionally, feeling embedded in the school community can enhance the perception of contributing to a larger goal of student success, further increasing work meaningfulness (Ghosh and Gurunathan, 2015; Rosso et al., 2010). Ultimately, when teachers experience work meaningfulness, they are likely to report higher levels of well-being (Steger et al., 2012).

The fourth hypothesis (H4) posits that work meaningfulness mediates the relationship between teacher collective efficacy and psychological well-being. Collective efficacy can contribute to work meaningfulness by fostering a sense of shared purpose and accomplishment in achieving educational goals (Donohoo et al., 2018; Goddard et al., 2015). When teachers believe they can collectively influence student learning outcomes (Goddard et al., 2004), they are more likely to perceive their work as valuable and significant (Dağdeviren Ertaş and Özdemir, 2024; Rosso et al., 2010). This perception of work meaningfulness, in turn, can lead to greater teacher well-being (Lavy and Naama-Ghanayim, 2020; Steger et al., 2012).

To further enrich the research and gain deeper insights into these relationships, the study incorporated a qualitative phase through digital ethnography. This phase aimed to capture the nuanced experiences and perspectives of Chinese teachers within online teaching communities. By engaging with teachers in their natural online environments, the qualitative phase sought to provide a richer, more contextualized understanding of how job embeddedness, collective efficacy, and work meaningfulness influence psychological well-being. The qualitative findings are expected to complement and expand upon the quantitative results, offering a comprehensive view of the complex interplay between these factors. This mixed-methods approach allows for a more robust examination of the research questions, providing both statistical validation and deep, contextual insights. Ultimately, the study aims to inform strategies for promoting teacher well-being within the Chinese educational system, offering valuable guidance for school leaders and policymakers.

## Methods and materials

### Participants and procedures

This study examined the influence of teacher job embeddedness, teacher collective efficacy, and work meaningfulness on the psychological well-being of Chinese teachers. A total of 406 in-service Chinese teachers participated in this study. Participants were recruited through a stratified random sampling method. Public and private schools across five provinces, representing different regions of China (East, West, South, North, and Central), were selected. Within each province, a list of Chinese schools was obtained from the local education bureau. Schools were then randomly chosen using a computer-generated random number list, ensuring proportional representation across regions. Teachers from these selected schools who met the inclusion criteria (being a full-time Chinese teacher for at least 1 year) were invited to participate (see Table 1 for participant demographics).

**Table 1:** Participant demographics.

Demographic characteristic	Mean (M) or number (%)	Standard deviation (SD)
Age	35.1 years	5.8 years
Gender		
Male	122 (30%)	
Female	284 (70%)	
Teaching experience	7.9 years	4.1 years
Educational background		
Bachelor’s degree	227 (56%)	
Master’s degree or higher	179 (44%)	

The study adhered to ethical research principles. All participants received an informed consent form presented at the beginning of the online survey. This form explained the study’s purpose, data confidentiality measures, and the right to withdraw participation at any point. Participation was voluntary, and no incentives were offered.

An online survey platform, Wenjuanxing, was selected for data collection due to its widespread usage among Chinese educators and its secure data encryption features. Wenjuanxing is a well-established tool in China that allows for efficient distribution of surveys, ensuring confidentiality and data security. The survey instruments employed validated measures, which were rigorously translated and back-translated from Chinese to English to ensure linguistic and conceptual accuracy. These instruments were designed to assess key variables: teacher job embeddedness, teacher collective efficacy, work meaningfulness, and psychological well-being. The data collection period lasted for 4 weeks, and to ensure participant confidentiality, all responses were coded and anonymized.

To gain deeper insights and complement the survey data, a digital ethnography was conducted within two prominent online teaching communities in China. The ethnography targeted two key online communities frequented by a large number of teachers. The first platform, with over 1.2 million registered users, facilitates discussions on curriculum resources, teaching methodologies, and educational policy updates. It is particularly known for its collaborative environment, where teachers actively share best practices and provide support to one another. The second platform, while slightly smaller with a community of over 800,000 teachers, focuses on challenges faced by educators in the Chinese school system, including workload management, student behavior issues, and administrative pressures. This community also hosts discussions on teacher well-being and strategies for maintaining motivation and mental health.

These online communities were chosen for their exclusive focus on Chinese teachers in China, ensuring that the discussions directly addressed the research context. Both platforms exhibited a high level of participation, with frequent posts and ongoing discussions providing rich data for analysis. Additionally, a preliminary analysis of forum topics revealed a prevalence of discussions related to job satisfaction, collaboration among teachers (collective efficacy), and finding meaning in their work (work meaningfulness), all of which are directly connected to the research focus.

To ensure ethical conduct throughout the study, several measures were implemented. Participants were assured of complete anonymity. Pseudonyms were used for all teacher quotes and interview transcripts, and a moderator-approved message was posted within each online community explaining the research purpose, data collection methods, and the participants’ right to choose participation and withdraw at any point. Additionally, all collected data, including field notes and interview transcripts, were securely stored on a password-protected university server to ensure data security.

While online forums offer a valuable window into teachers’ experiences, it is acknowledged that participation might be skewed towards specific viewpoints. Teachers who are particularly passionate or dissatisfied with their work experiences might be more likely to engage in online discussions. To mitigate this potential bias and enhance the generalizability of the qualitative findings, the study employed several strategies. First, a purposive sampling approach was used to recruit participants from the online communities. This involved actively seeking out teachers with diverse experiences and perspectives on job embeddedness, collective efficacy, and work meaningfulness. For instance, the recruitment message could highlight the desire to hear from teachers at different stages of their careers, in various grade levels, or from different school environments (urban, rural). Second, during semi-structured interviews, open-ended topic prompts and probing questions aimed to elicit a wider range of perspectives beyond those initially presented by participants. This could involve encouraging teachers to elaborate on their experiences, share both positive and negative aspects of their work environment, or offer suggestions for improving teacher well-being in their schools. Finally, data collection continued until data saturation was achieved, indicating that no new themes or insights were emerging from the interviews. This ensured that a comprehensive range of perspectives were captured within the qualitative data.

### Translation procedures and pilot testing

This study employed established instruments to measure the key constructs: job embeddedness, collective teacher efficacy, perceived work meaningfulness, and psychological well-being. To ensure the instruments’ cultural appropriateness and maintain the integrity of the original measures, a multi-pronged approach was adopted.

First, validated and reliable instruments were chosen whenever possible. For instance, the Collective Teacher Efficacy scale developed by Tschannen-Moran and Barr (2004) has been previously validated for use in the Chinese educational context by Lee et al. (2011). For instruments that lacked pre-existing validated Chinese translations, we employed a rigorous translation and back-translation process. Translation was conducted by two bilingual professionals with expertise in both English and Mandarin Chinese and a strong background in educational psychology. One translator carried out the forward translation from English to Chinese, followed by a separate translator conducting the back-translation to English. The two versions were compared to ensure semantic equivalence, and any discrepancies were discussed and resolved with input from both translators. This process ensured that the final Chinese version was culturally relevant and maintained the original meaning of the scales.

Following translation and back-translation, a pilot test was conducted with 40 Chinese teachers who were not included in the final sample. The purpose of the pilot was twofold: to assess the clarity of the translated instruments and to evaluate the reliability and validity of the scales within the Chinese context. Participants provided feedback on the comprehensibility of the items, particularly regarding any cultural nuances that might affect understanding. Pilot data were analyzed using descriptive statistics, and reliability estimates (Cronbach’s alpha) were computed. Based on feedback, minor adjustments were made to improve item clarity, particularly in the “organizational fit” and “work meaningfulness” items, which required rewording to better align with the Chinese educational context.

### Job embeddedness

The evaluation of job embeddedness was conducted using a selection from the Job Embeddedness Scale by Mitchell et al. (2001), with a translation provided by Wang (2020). Out of the original 40-item scale, 14 items were carefully chosen to measure the extent of nurses’ embeddedness in their job roles. The scale is divided into three subscales: organizational links, organizational fit, and organizational sacrifice, featuring items such as “I maintain close relationships with my work colleagues,” “I perceive myself as a good match for my current position,” and “Leaving my job would significantly disrupt my life and that of my family,” respectively. These items were assessed on a 5-point Likert scale, where 1 signifies ‘strongly disagree’ and 5 ‘strongly agree.’ In this study, the scale demonstrated strong internal consistency, with Cronbach’s alpha for job embeddedness recorded at 0.82. Construct validity was tested through Confirmatory Factor Analysis (CFA), with the following model fit indices: RMSEA = 0.065, SRMR = 0.041, CFI = 0.925, and TLI = 0.902, all indicating acceptable fit. These values confirm that the scale is appropriate for use within this context.

### Perceived work meaningfulness

The assessment of perceived work meaningfulness incorporated five items drawn from the work of Roh and Suh (2014), pinpointing the significance individuals attribute to their work. Four of these items were adapted from the Meaning at Work framework by Ashmos and Duchon (2000), for instance, “The work I do is intertwined with my core life values.” An additional item, sourced from Kinjerski and Skrypnek (2006), emphasizes the congruence between work demands and the individual’s personal values, beliefs, and behaviors. The scale showed acceptable internal consistency, with a Cronbach’s alpha of 0.79. Construct validity fit indices from CFA were also acceptable: RMSEA = 0.062, SRMR = 0.038, CFI = 0.910, and TLI = 0.899, indicating that the items captured the construct well within this sample.

### Collective teacher efficacy

To gauge collective teacher efficacy, the study employed the scale developed by Tschannen-Moran and Barr (2004), which aligns with Bandura’s (1997) conceptual guidelines. This scale assesses teachers’ collective beliefs regarding their school’s efficacy in instructing students and fostering academic achievement. It encompasses two dimensions, instructional strategies and student discipline, each measured by six items. Teachers responded to these items on a 5-point scale, from ‘nothing’ to ‘a great deal.’ The instrument has been validated for use in the Chinese educational context, notably among teachers in Hong Kong (Lee et al., 2011). In the current study, the reliability of the collective efficacy scale was strong, with a Cronbach’s alpha of 0.83. CFA results further validated the scale’s appropriateness, with fit indices as follows: RMSEA = 0.068, SRMR = 0.044, CFI = 0.920, and TLI = 0.898. These values indicate a good fit for the model, suggesting that collective efficacy is effectively measured in the Chinese educational context.

### Psychological well-being

Participants’ psychological well-being was assessed using the Psychological Well-Being Scale by Ryff and Keyes (1995). This 18-item scale measures six aspects of well-being: positive relations with others, personal growth, self-acceptance, purpose in life, autonomy, and environmental mastery. Responses were given on a seven-point Likert scale, ranging from ‘strongly disagree’ to ‘strongly agree.’ The scale’s broad applicability and reliability have been well-documented in previous studies. In the current study, Cronbach’s alpha for this scale was 0.84, indicating high internal consistency. Construct validity for this scale was supported through CFA, with fit indices showing RMSEA = 0.059, SRMR = 0.039, CFI = 0.915, and TLI = 0.908, all of which suggest the model fit the data reasonably well.

### Qualitative data collection

The qualitative phase of this study utilized a digital ethnography approach, enabling an in-depth exploration of teachers’ experiences related to job embeddedness, collective efficacy, and work meaningfulness within online communities. Digital ethnography, a well-established method for studying online cultures (Hine, 2000), provided a naturalistic setting for observing and engaging with participants in an authentic context. Over a period of 4 months, the researcher actively participated in two established online communities frequented by Chinese teachers. These communities were selected based on their relevance to education and their active engagement on topics related to teacher well-being and professional challenges.

To maintain ethical rigor, the researcher adopted a professional and non-intrusive role in the discussions, being mindful not to influence the natural flow of conversations. The researcher operated under an observational-participatory mode, meaning that while the researcher contributed to discussions, the focus was on facilitating insights rather than dominating the discourse. This strategy helped ensure that the data collected remained reflective of participants’ authentic experiences. Informed consent was obtained through private messages when participants agreed to partake in the interview phase of the study.

The researcher used a combination of passive and active engagement techniques. Passive engagement involved monitoring and documenting ongoing discussions, focusing on naturally emerging themes relevant to the research questions. Active engagement included strategically posting open-ended questions to elicit deeper reflections from participants on their experiences with job embeddedness, collaboration, and meaning in their work. This mixed approach enabled the researcher to capture both organic, unsolicited perspectives and more detailed, reflective insights on the research themes.

Several techniques were employed to ensure comprehensive data collection. Detailed field notes were taken during each interaction, capturing both the content of discussions and the emotional undertones communicated through participants’ language choices, including expressions of frustration, satisfaction, and camaraderie. Non-verbal cues, such as the use of emojis, reaction gifs, and other digital markers, were also noted as they provided contextual clues about the emotional climate of the discussions. The combination of textual and visual data enriched the dataset, allowing for a more nuanced understanding of the participants’ experiences.

In addition to observational data, semi-structured interviews were conducted with 10 active participants identified from the online discussions. The interview protocol was designed to explore the key themes of job embeddedness, collective efficacy, and work meaningfulness in greater depth. The interviews were conducted via video conferencing software to accommodate participants’ preferences and to build rapport. Each interview was recorded, transcribed, and added to the corpus of qualitative data for analysis.

To ensure the appropriateness of the interview questions and engagement strategies, a pilot test was conducted with three participants from the online communities prior to the full data collection. Feedback from the pilot was used to refine the interview guide and improve the clarity of questions. Reflexivity was also a critical aspect of this study, with the researcher maintaining a reflexive journal throughout the data collection period. This journal documented any potential biases, assumptions, or changes in the researcher’s perspective, which helped mitigate the influence of researcher subjectivity on the data interpretation.

### Data analysis

The data analysis process commenced with preliminary screening and descriptive analyses conducted using the statistical software package SPSS 28. Subsequently, a two-stage structural equation modeling (SEM) approach, facilitated by AMOS 26 (Kline, 2015), was employed to explore the hypothesized relationships between the study variables. In the first stage, emphasis was placed on evaluating the reliability and validity of the measurement instruments. This involved assessing the internal consistency (reliability) of each scale using Cronbach’s alpha coefficient. Additionally, scrutiny was placed on the factor loadings of individual items to ensure their adequate representation of the respective latent constructs (validity). Once the measurement model was deemed acceptable, attention shifted to the second stage, focusing on testing the hypothesized relationships between the latent variables within the proposed model. Here, a range of fit indices including the root-mean-square error of approximation (RMSEA), standardized root mean square residual (SRMR), chi-square/degree of freedom ratio, comparative fit index (CFI), goodness-of-fit index (GFI), and Tucker-Lewis index (TLI) were utilized to assess the overall model fit. These indices, with established cut-off values (Browne and Cudeck, 1993; Kline, 2015; Vandenberg and Lance, 2000), were instrumental in determining the acceptability of the model fit.

The data collected through field notes, interviews, and online interactions were analyzed using thematic analysis (Braun and Clarke, 2006). The analysis followed a systematic process, beginning with data familiarization, where the researcher repeatedly read the field notes and interview transcripts to gain a deep understanding of the content. During the open coding phase, segments of the text were assigned descriptive codes that captured key ideas and concepts. The researcher employed NVivo software to assist in organizing and managing the vast amount of qualitative data.

Following the initial coding, axial coding was used to identify patterns and relationships between the codes, resulting in the formation of broader thematic categories. Themes such as “supportive school culture,” “teacher collaboration,” and “work-life balance” emerged from this process, reflecting the core areas of job embeddedness, collective efficacy, and work meaningfulness. The constant comparison method (Glaser and Strauss, 1967) was applied to ensure that the themes were consistent across different data sources, including field notes and interview transcripts.

To enhance the credibility of the findings, member checking was employed. A subset of the participants was asked to review the preliminary themes and provide feedback on whether they accurately represented their experiences. Their input was used to refine and finalize the themes, adding another layer of rigor to the analysis. Triangulation was also achieved by integrating the qualitative findings with the quantitative data from the structural equation modeling (SEM) analysis. This allowed for a richer, more holistic interpretation of the results. For example, the quantitative findings on the mediating role of work meaningfulness were corroborated by qualitative data highlighting teachers’ reflections on how their sense of purpose contributed to their well-being.

The study employed multiple strategies to ensure the trustworthiness of the qualitative findings. In addition to member checking and triangulation, an audit trail was maintained, documenting the research process from data collection to analysis. This provided transparency in the methodological choices and ensured that the study could be replicated or assessed for reliability. Peer debriefing sessions were also conducted with fellow researchers to critically assess the coding process and theme development, further ensuring the dependability of the findings.

## Results

### Quantitative results

#### Preliminary data analysis

Prior to conducting the main analyses, a series of preliminary data checks were performed to ensure the data quality and suitability for SEM (Hair et al., 2010). These checks addressed missing values, normality of data distribution, and the presence of potential outliers.

Missing data can potentially introduce bias and reduce the accuracy of statistical tests (Tabachnick and Fidell, 2007). The extent of missing data was assessed for each variable. If missing values were minimal (less than 5%), list wise deletion, a conservative approach that removes cases with any missing data, was considered appropriate (Hair et al., 2010). However, if missing data exceeded 5%, missing data imputation techniques such as mean imputation or expectation–maximization (EM) could be explored to retain valuable data points while minimizing potential bias.

Univariate normality was assessed for each observed variable using skewness and kurtosis statistics, along with visual inspection of histograms and Q-Q plots. If significant deviations from normality were detected, data transformations (e.g., square root or log transformation) could be employed to achieve a more normal distribution. Additionally, multivariate normality, referring to the normality of the distribution of error terms in the model, was evaluated using Mardia’s test (Mardia, 1972). While slight violations of multivariate normality may be tolerable with larger sample sizes, significant deviations might necessitate the use of robust statistics or alternative SEM estimators. Also, outliers were identified through examination of boxplots and standardized scores (z-scores). Depending on the severity and number of outliers, decisions could be made to winsorize the data (replacing extreme values with the nearest non-outlier) or conduct sensitivity analyses to assess the impact of outliers on the overall results (Tabachnick and Fidell, 2007).

#### Construct validity

Confirmatory factor analysis (CFA) was conducted to assess the hypothesized measurement model’s validity. The resulting fit indices indicated an acceptable model fit: χ^2^ = 250.23, χ^2^/df = 2.09, *p* < 0.001; RMSEA = 0.064; SRMR = 0.042; CFI = 0.927; TLI = 0.901. The overall fit indices provided support for the hypothesized measurement model. The observed variables adequately represent their underlying latent constructs (Teacher Job Embeddedness, Teacher Collective Efficacy, Work Meaningfulness, and Psychological Well-being), and the model provides a reasonable fit to the data. This suggests that we can proceed with further analysis to test the hypothesized relationships between these constructs.

Table 2 presents the descriptive statistics and correlations for the study variables. All variables were measured on a scale ranging from 1 (strongly disagree) to a higher score indicating greater agreement. The mean scores (M) for the four constructs ranged from 3.58 (work meaningfulness) to 4.23 (job embeddedness), with standard deviations (SD) between 0.68 (psychological well-being) and 0.92 (work meaningfulness). These results suggest that, on average, teachers reported moderate levels of job embeddedness, collective efficacy, and psychological well-being, with work meaningfulness scoring slightly lower.

**Table 2:** Descriptive statistics and correlations of study variables.

Construct	M (SD)	1	2	3	4
1. Job embeddedness	4.23 (0.87)	1.00	–		
2. Collective efficacy	3.81 (0.74)	0.32	1.00		
3. Work meaningfulness	3.58 (0.92)	0.28	0.41	1.00	
4. Psychological well-being	3.92 (0.68)	0.17	0.24	0.37	1.00

The correlation coefficients between the study variables are displayed in the upper right triangle of the table. Job embeddedness exhibited positive correlations with both collective efficacy (*r* = 0.32) and psychological well-being (*r* = 0.17). These correlations indicate that teachers with higher reported job embeddedness tended to report slightly higher levels of collective efficacy and psychological well-being. Work meaningfulness also demonstrated positive correlations with both collective efficacy (*r* = 0.41) and psychological well-being (*r* = 0.37), suggesting that teachers who perceived greater meaning in their work also reported higher levels of collective efficacy and well-being. The strength of the correlation between work meaningfulness and the other two variables is somewhat stronger than the correlation between job embeddedness and the other two variables.

#### Internal consistency and convergent validity

Table 3 presents the results for the assessment of internal consistency (reliability) and convergent validity of the measurement model. Internal consistency refers to the extent to which the observed variables within a construct measure the same underlying concept. Convergent validity assesses the degree to which the observed variables converge to represent their designated latent construct.

**Table 3:** Reliability and convergent validity of the constructs.

Metric	Job embeddedness	Collective efficacy	Work meaningfulness	Well-being
AVE	0.68	0.62	0.54	0.50
MSV	0.47	0.42	0.37	0.34
ASV	0.16	0.13	0.10	0.09
Cronbach’s α/CR	0.82	0.78	0.75	0.72

The table displays the Average Variance Extracted (AVE), Maximum Shared Variance (MSV), Average Shared Variance (ASV), and Cronbach’s alpha (*α*) / Composite Reliability (CR) for each construct. The AVE reflects the amount of variance in the indicators captured by their underlying latent construct. In this study, all AVE values (ranging from 0.50 for psychological well-being to 0.68 for job embeddedness) exceed the recommended benchmark of 0.50 (Hair et al., 2010), indicating that at least half of the variance in the observed measures is explained by their respective latent constructs. This suggests good convergent validity.

Composite Reliability (CR) provides a more accurate estimate of internal consistency reliability compared to Cronbach’s alpha, particularly in models with smaller sample sizes (Hair et al., 2010). Here, all CR values are greater than their corresponding AVE values, further supporting the internal consistency and convergent validity of the measurement model.

Discriminant validity ensures that the latent constructs in the model are distinct from each other. This is established when the variance shared between any two constructs (measured by MSV and ASV) is lower than the variance unique to each construct (measured by AVE) (Hair et al., 2009). As shown in Table 3, all MSV and ASV values (ranging from 0.09 to 0.16) are lower than their corresponding AVE values (ranging from 0.50 to 0.68). This confirms that the constructs share less variance with each other compared to the variance they explain individually, providing evidence for discriminant validity.

Harman’s one-factor test was conducted to assess the potential for common method bias, which can arise when using self-reported data for all constructs. This test involves conducting an exploratory factor analysis (EFA) with all observed variables from the model. If a single factor emerges that explains a large proportion of the variance (typically exceeding 50%), it suggests that a common method bias might be present (Podsakoff and Organ, 1986).

In this study, Harman’s one-factor test revealed that the first factor explained 41.7% of the variance in the data. This value falls below the recommended threshold of 50%, suggesting that common method bias is unlikely to be a significant concern in this analysis.

Therefore, based on the Harman’s one-factor test results, we can be cautiously optimistic that common method bias is not a major threat to the validity of the findings. However, it is important to acknowledge that this is just one test, and other methods for detecting common method bias could be explored for further confirmation.

### Structural model results

**Figure 1:**
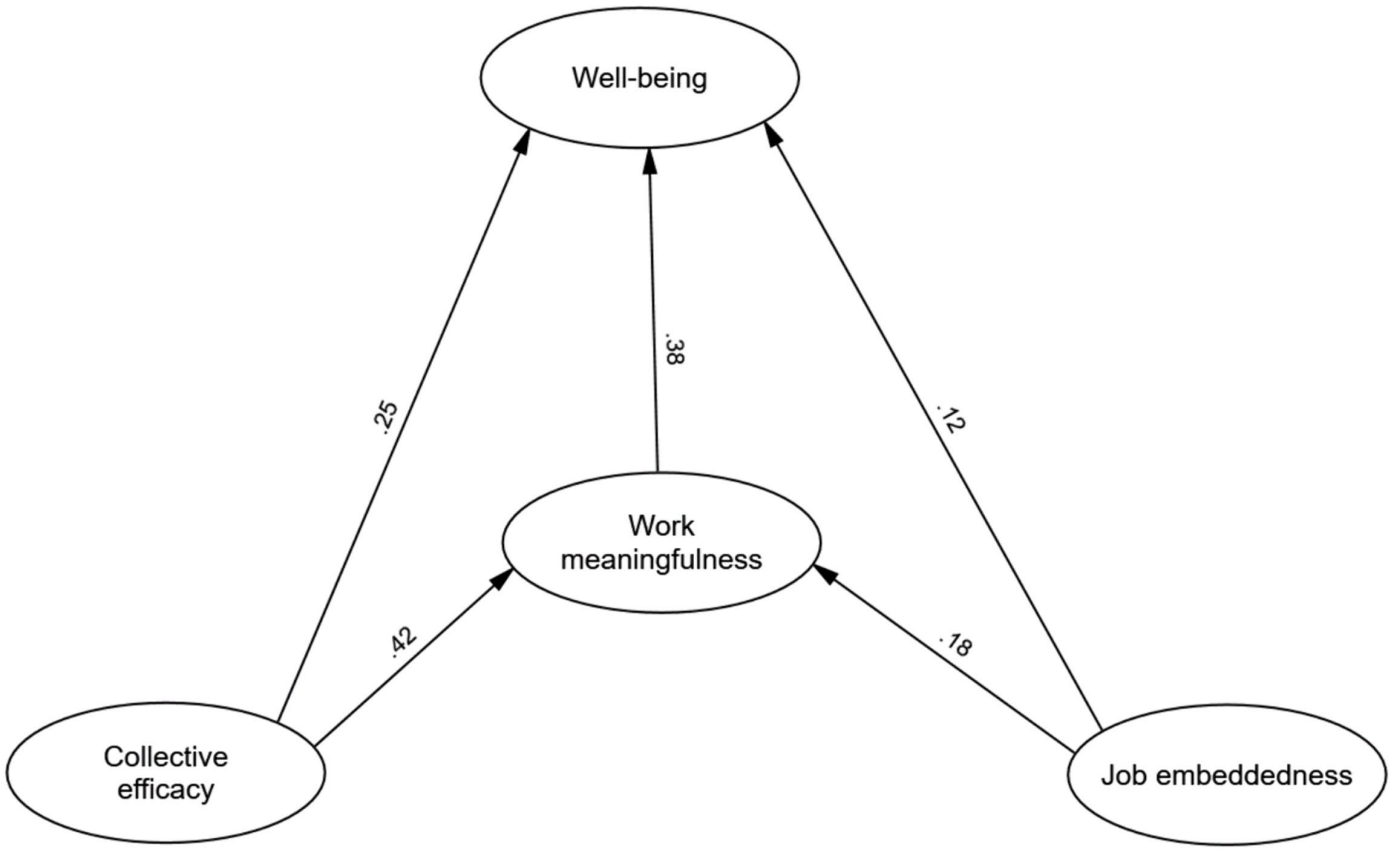
The model of interplay of job embeddedness, collective efficacy, and work meaningfulness on teacher well-being.

The hypothesized structural model was tested using AMOS software. The resulting fit indices indicated an acceptable model fit: χ^2^ = 287.12, df = 102, χ^2^/df = 2.81, *p* < 0.001; RMSEA = 0.072; SRMR = 0.048; CFI = 0.914; TLI = 0.889. The path coefficients were all significant, as depicted in Figure 1.

Table 4 presents the path coefficients (*β*) and standardized estimates for the hypothesized relationships between the study variables. Standardized estimates, reported with 95% confidence intervals (CI), represent the change in one standard deviation in the predictor variable associated with a one standard deviation change in the criterion variable, controlling for other variables in the model.

**Table 4:** Path coefficients and standardized estimates.

Path	Direct effects β	95% CI	Indirect effects β	95% CI	Total effects β	95% CI
Embeddedness → Well-being	0.12	(0.02, 0.14)	0.07	(0.03, 0.11)	0.19	(0.09, 0.21)
Efficacy → Well-being	0.25	(0.13, 0.27)	0.16	(0.10, 0.20)	0.41	(0.32, 0.44)
Meaningfulness → Well-being	0.38	(0.26, 0.40)	–	–	–	–
Embeddedness → Meaningfulness	0.18	(0.22, 0.08)	–	–	–	–
Efficacy → Meaningfulness	0.42	(0.32, 0.44)	–	–	–	

Embeddedness: job embeddedness; Efficacy: collective efficacy; Meaningfulness: work meaningfulness.

The results reveal significant positive direct effects of all three focal constructs on psychological well-being. Job embeddedness had a small but significant direct effect on well-being (*β* = 0.12, estimate = 0.08, CI = [0.02, 0.14]), indicating that teachers with higher reported job embeddedness tended to report slightly higher levels of psychological well-being. Collective efficacy had a moderate positive direct effect on well-being (*β* = 0.25, estimate = 0.19, CI = [0.13, 0.27]), suggesting that teachers who perceived a stronger collective belief in their school’s ability to impact student learning also reported higher levels of well-being. Work meaningfulness exhibited the strongest direct effect on well-being (*β* = 0.38, estimate = 0.32, CI = [0.26, 0.40]), indicating that teachers who found greater meaning in their work reported the highest levels of psychological well-being.

Job embeddedness also had a small but significant direct effect on work meaningfulness (*β* = 0.18, estimate = 0.15, CI = [0.02, 0.22]), suggesting that teachers with stronger connections to their workplace also reported a slightly higher sense of meaning in their work. The strongest direct effect was observed between collective efficacy and work meaningfulness (*β* = 0.42, estimate = 0.38, CI = [0.32, 0.44]), indicating that teachers who perceived a stronger collective belief in their school’s effectiveness also reported a greater sense of meaning in their work.

The model also revealed significant indirect effects of job embeddedness and collective efficacy on psychological well-being, mediated by work meaningfulness. The indirect effect of job embeddedness on well-being through work meaningfulness was small but significant (*β* = 0.07, estimate = 0.07, CI = [0.03, 0.11]). Similarly, the indirect effect of collective efficacy on well-being through work meaningfulness was also significant (*β* = 0.16, estimate = 0.15, CI = [0.10, 0.20]). These findings suggest that work meaningfulness partially mediates the relationship between both job embeddedness and collective efficacy with psychological well-being.

The total effects represent the combined influence (direct and indirect) of a predictor variable on a criterion variable. The total effect of job embeddedness on well-being was small but significant (*β* = 0.19, estimate = 0.15, CI = [0.09, 0.21]). Collective efficacy had a moderate total effect on well-being (*β* = 0.41, estimate = 0.38, CI = [0.32, 0.44]), suggesting that it is the strongest predictor of psychological well-being among the three focal constructs in this study.

### Qualitative results

The digital ethnography conducted within the two online communities yielded rich qualitative data that complemented the quantitative findings from the initial survey. The thematic analysis of field notes and interview transcripts revealed several key themes related to job embeddedness, collective efficacy, and work meaningfulness among Chinese teachers.

A strong theme emerged around the importance of feeling connected to a supportive school community in fostering job embeddedness. Teachers who described positive relationships with colleagues and strong administrative leadership expressed a greater sense of job embeddedness. For instance, one teacher (Li Wei, pseudonym) from a smaller rural school shared, “Knowing that my principal trusts me and that I can rely on my fellow teachers for help makes a huge difference. It feels like we are all in this together.” Additionally, the feeling of being invested in student success was another significant factor contributing to job embeddedness. Teachers who felt their efforts positively impacted student learning and development expressed a stronger sense of purpose and connection to their work. A high school physics teacher (Wang Lin, pseudonym) highlighted this in his interview, stating, “The most rewarding moments are when students finally grasp a difficult concept. Knowing I played a role in their success makes coming to work every day worthwhile.”

However, the challenge of maintaining a healthy work-life balance emerged as a potential barrier to job embeddedness. Teachers overwhelmed by administrative tasks, lengthy working hours, and excessive workloads reported feeling burnt out and disconnected from their jobs. A middle school English teacher (Zhang Yue, pseudonym) expressed this sentiment, “The endless paperwork and late nights grading take away from the time I could spend with my family. It makes it hard to feel truly invested in my work at school.”

In terms of collective efficacy, teachers who perceived strong collaboration and teamwork among colleagues within their schools exhibited a higher sense of collective efficacy. Sharing resources, planning lessons collaboratively, and supporting each other through challenges were identified as key contributors to this feeling. A mathematics teacher (Sun Mei, pseudonym) from a large urban school described, “Our math department has weekly meetings where we discuss best practices and support each other. Knowing I can learn from my colleagues and that they have my back makes a big difference.” The role of supportive school leadership in fostering collective efficacy was also evident. Teachers who felt their principals valued their input, encouraged professional development, and addressed their concerns reported a stronger belief in their collective ability to improve student outcomes. A primary school teacher (Liu Hao, pseudonym) shared, “Our principal is always open to our suggestions and makes sure we have access to professional development opportunities. This makes us feel valued and confident in our ability to make a positive impact.”

Conversely, a lack of collaboration and a competitive environment among teachers emerged as barriers to collective efficacy. Teachers in schools where individual performance was emphasized over teamwork reported feeling isolated and unsure of their collective ability to achieve shared goals. A high school biology teacher (Chen Jie, pseudonym) described this challenge, stating, “There’s not much collaboration in our department. Everyone seems focused on their own classes, and there’s little communication or sharing of resources. It makes it hard to feel like we are working towards a common goal.”

Regarding work meaningfulness, the opportunity to witness student engagement and academic achievement was a significant factor contributing to a sense of meaningfulness. Teachers who felt their efforts directly impacted student learning and development reported a greater sense of purpose and fulfillment in their work. An elementary school teacher (Xu Li, pseudonym) highlighted this, stating, “There’s nothing more rewarding than seeing the ‘light bulb’ moment when a student finally understands a concept. Knowing I play a role in shaping their future makes my job incredibly meaningful.” Teachers who felt their work aligned with their personal values, such as a passion for a particular subject or a desire to help others, reported a stronger sense of meaningfulness. A history teacher (Wang Dong, pseudonym) explained, “I’ve always been fascinated by history, and I love sharing that passion with my students. Helping them understand the past and connect it to the present is what makes my job so meaningful.”

However, bureaucratic constraints and a focus on standardized testing were reported as diminishing the sense of work meaningfulness. Teachers expressed frustration that these factors limited their ability to implement creative teaching methods and cater to the individual needs of their students. A middle school English teacher (Zhao Ming, pseudonym) described this challenge, stating, “The pressure to prepare students for standardized tests takes away the joy of teaching. We have less time to explore interesting topics in depth or cater to individual student needs. It makes it hard to feel like I’m making a real difference in their lives.”

The themes identified through the digital ethnography resonate with the quantitative findings from the initial survey. For instance, the importance of a supportive school community and strong administrative leadership emerged as factors contributing to both job embeddedness and collective efficacy. Similarly, the challenge of maintaining a healthy work-life balance and the pressure of standardized testing emerged as potential barriers to both job embeddedness and work meaningfulness. These qualitative findings provide valuable context and deeper understanding to the quantitative results, illustrating the lived experiences of Chinese teachers and the specific factors that influence their sense of job embeddedness, collective efficacy, and work meaningfulness.

## Discussion

The present study investigated the relationships between teacher job embeddedness, teacher collective efficacy, work meaningfulness, and psychological well-being among Chinese teachers, providing valuable insights that contribute to the growing body of research on factors influencing teacher well-being. The findings from both the quantitative and qualitative phases offer a comprehensive understanding of these relationships, reinforcing existing theories and highlighting new areas for consideration.

The first hypothesis, which posited a direct positive relationship between teacher job embeddedness and psychological well-being, was supported. This aligns with previous research suggesting that feeling embedded within the workplace fosters a sense of security, belonging, and social support (Ahmad et al., 2023; Burrows et al., 2022; Chan et al., 2019; Feldman et al., 2012; Khairunisa and Muafi, 2022; Mitchell et al., 2001; Steindórsdóttir et al., 2020; Sun et al., 2012). In the context of Chinese culture, where collectivism and social connectedness are highly valued (Du and Wei, 2015), strong on-the-job relationships and a sense of community within the school likely contribute significantly to teacher well-being. It is plausible that in a culture where interpersonal relationships and group harmony are highly valued, the sense of being integrated and connected within the school community plays a particularly vital role in promoting teacher well-being. Furthermore, our qualitative data lends support to this interpretation. The narratives of teachers like Li Wei, who emphasized the importance of trust and support from colleagues and administration, highlight the crucial role of strong social connections in fostering a sense of embeddedness and, consequently, enhancing well-being. This suggests that school leaders in China may be particularly effective in promoting teacher well-being by actively cultivating a supportive and inclusive school culture that encourages collaboration, mutual respect, and open communication.

The second hypothesis, proposing a direct positive relationship between teacher collective efficacy and psychological well-being, was also confirmed. This finding aligns with prior studies highlighting the positive influence of collective efficacy on teacher well-being (Capone et al., 2018; Dağdeviren Ertaş and Özdemir, 2024; Herrera et al., 2022; Strahannée Brown et al., 2019). Schools characterized by high collective efficacy foster supportive and positive climates (Fathi et al., 2020; Liang et al., 2022), where teachers feel a sense of shared belief in their ability to impact student learning (Donohoo, 2017). The shared belief in a school’s collective ability to impact student learning appears to foster a sense of purpose and empowerment among teachers, contributing to their overall well-being. However, our study goes a step further by suggesting that the impact of collective efficacy on well-being may be particularly pronounced in the Chinese context. The qualitative insights from teachers like Sun Mei, who highlighted the value of regular collaborative meetings, suggest that the opportunity to work together towards shared goals and contribute to the collective success of the school resonates strongly with the collectivist values prevalent in Chinese culture.

School leaders can cultivate collective efficacy by implementing practices that encourage teacher collaboration and shared decision-making (Loughland and Ryan, 2022). This might involve including teachers in discussions about school goals and improvement initiatives. Furthermore, celebrating successes achieved through collective effort can further strengthen teachers’ belief in their collective ability to make a positive impact (Tschannen-Moran and Barr, 2004).

The third hypothesis, which proposed that work meaningfulness mediates the relationship between teacher job embeddedness and psychological well-being, received support. This finding suggests that job embeddedness fosters work meaningfulness in several ways. As the study participants were Chinese teachers, the strong on-the-job connections facilitated by job embeddedness may have provided opportunities for collaboration and knowledge sharing, which can contribute to a sense of purpose and the value of one’s work (Bailey and Madden, 2016; Marasi et al., 2016). Additionally, feeling embedded in the school community can enhance the perception of contributing to a larger goal of student success, further increasing work meaningfulness (Ghosh and Gurunathan, 2015; Rosso et al., 2010). Ultimately, when teachers experience work meaningfulness, they are likely to report higher levels of well-being (Arnold et al., 2007; Crego et al., 2020; Görgens-Ekermans and Steyn, 2016; Hooker et al., 2020). Our results suggest that job embeddedness, characterized by strong on-the-job connections and a sense of belonging within the school community, fosters work meaningfulness, which in turn contributes to enhanced psychological well-being.

The qualitative data gathered through digital ethnography provides compelling evidence to support this interpretation. The narratives of teachers like Xu Li, who found deep meaning in witnessing the positive impact of their teaching on student learning, highlight the intrinsic rewards that can be derived from the teaching profession. These findings challenge the notion that work meaningfulness is solely dependent on external factors such as salary or recognition, suggesting that the inherent value of contributing to student growth and development can be a powerful source of meaning for teachers.

However, it is important to note that our findings may differ from those in Western contexts, where individualism and personal achievement are often emphasized. In contrast, the collectivist nature of Chinese culture (Du and Wei, 2015) might explain why job embeddedness, with its emphasis on social connections and shared goals, plays such a crucial role in fostering work meaningfulness among Chinese teachers. This suggests that interventions aimed at promoting teacher well-being in China should consider the cultural context and prioritize strategies that strengthen social bonds and collaborative efforts within the school community.

The fourth hypothesis, proposing that work meaningfulness mediates the relationship between teacher collective efficacy and teacher psychological well-being, was also supported. This finding suggests that collective efficacy contributes to work meaningfulness by fostering a sense of shared purpose and accomplishment in achieving educational goals (Donohoo et al., 2018). When teachers believe they can collectively influence student learning outcomes (Bandura, 1997), they are more likely to perceive their work as valuable and significant (Hirschi, 2012; Rosso et al., 2010). This perception of work meaningfulness, in turn, can lead to greater teacher well-being (Arnold et al., 2007; Crego et al., 2020; Golparvar and Abedini, 2014; Han et al., 2021; Steger et al., 2012).

However, while these findings are consistent with the literature, they also challenge the assumption that collective efficacy’s effect on well-being is solely direct. Our results suggest that the relationship is more complex, with work meaningfulness serving as a crucial intermediary. This implies that collective efficacy may not always directly improve well-being but does so by enhancing how meaningful teachers perceive their work to be. This insight builds on Steger et al. (2012) and offers a more nuanced understanding of how collective efficacy functions within educational settings. It emphasizes that simply fostering collective efficacy without addressing the meaningfulness of work may result in less impactful improvements in teacher well-being.

Moreover, our findings extend the conversation into the cultural realm. In the context of China’s collectivist culture, the significance of group-oriented achievement is deeply ingrained (Du and Wei, 2015). This cultural factor might amplify the role of collective efficacy in shaping work meaningfulness, as teachers derive not only personal satisfaction but also cultural validation from working towards collective educational goals. In contrast, in more individualistic contexts, the relationship between collective efficacy and work meaningfulness may not be as pronounced, as individual success and autonomy tend to be more highly valued (Ng and Feldman, 2010). Therefore, while collective efficacy is universally important, the cultural context may influence the extent to which it contributes to work meaningfulness and, consequently, well-being.

The qualitative phase of this study further enriches these findings, particularly by highlighting the complex interplay between job embeddedness, collective efficacy, and work meaningfulness. The digital ethnography revealed that a supportive school community is a key driver of both job embeddedness and collective efficacy. This aligns with existing literature that suggests that supportive work environments are essential for fostering teacher satisfaction and reducing turnover intentions (Khairunisa and Muafi, 2022; Steindórsdóttir et al., 2020). However, our study adds a critical dimension: the role of leadership in mediating these factors. Teachers like Li Wei, who felt supported by their colleagues and administration, exemplify how leadership creates a context in which embeddedness and collective efficacy can thrive. This finding corroborates Leithwood and Jantzi (2008), who argue that empowering leadership is fundamental to building collective efficacy. Yet, our results suggest that leadership’s influence may be even more profound in collectivist cultures, where hierarchical relationships often shape interpersonal dynamics more strongly than in individualistic contexts.

On the other hand, the qualitative data also pointed to barriers, particularly the challenge of maintaining a healthy work-life balance, which surfaced as a recurring theme. Teachers overwhelmed by administrative tasks and excessive workloads, such as Zhang Yue, reported feelings of burnout and disengagement. This is consistent with Skaalvik and Skaalvik's (2019) findings on the negative effects of high workloads on teacher well-being. Interestingly, this tension between professional responsibilities and personal life creates a paradox: while teachers feel embedded and connected through their work, the administrative burdens may undermine the very well-being that embeddedness seeks to protect. This raises critical questions about the limits of job embeddedness and whether there is a tipping point at which high embeddedness leads to role overload rather than well-being. Future research could explore this threshold more thoroughly, particularly in high-pressure educational environments like those in China.

Another key theme identified through the qualitative phase was the importance of work meaningfulness in sustaining teacher well-being. Teachers like Xu Li emphasized how witnessing student engagement and achievement contributed to a strong sense of purpose. This is consistent with Rosso et al. (2010), who argued that work meaningfulness is enhanced when employees perceive tangible outcomes from their efforts. However, our study contributes a new layer of understanding by exploring how work meaningfulness is shaped by collective values. Teachers in collectivist cultures may derive more meaning from their work when they perceive their efforts as contributing to the success of the broader educational community. This contrasts with more individualistic settings, where personal achievements may play a larger role in shaping work meaning.

Furthermore, teachers who felt that their work aligned with their personal values, like Wang Dong, reported a greater sense of fulfillment. This aligns with Bailey and Madden’s (2016) assertion that alignment between personal values and professional activities is a critical contributor to work meaningfulness. However, our findings suggest that value alignment may be more challenging to achieve in highly standardized educational systems, where teachers have limited autonomy over curriculum design and teaching methods. In such systems, even high levels of collective efficacy may not fully compensate for the lack of personal agency in shaping one’s work, a topic that merits further exploration.

In contrast, teachers in more flexible educational systems may experience higher levels of work meaningfulness, even in the absence of strong collective efficacy, simply because they have more control over how they execute their professional responsibilities. Therefore, while collective efficacy and job embeddedness are critical to teacher well-being, they may need to be complemented by autonomy-enhancing policies that allow teachers to align their professional work with their personal values.

Taken together, the findings of this study suggest that fostering collective efficacy and job embeddedness are essential but not sufficient conditions for enhancing teacher well-being. The mediating role of work meaningfulness offers critical insights into how schools can structure their environments to promote not just a sense of community and shared purpose but also the intrinsic value of teaching as a profession. This finding has practical implications for educational leaders, particularly in systems like China’s, where hierarchical structures and heavy workloads may undermine the potential benefits of embeddedness and collective efficacy if not managed carefully.

The study also underscores the importance of cultural context in shaping how these relationships play out. While job embeddedness, collective efficacy, and work meaningfulness are important across all contexts, their relative influence on teacher well-being may vary depending on the cultural and organizational structures within which teachers operate. Future research could extend this work by comparing these dynamics across different educational systems, particularly those in individualistic versus collectivist societies, to build a more comprehensive understanding of how to foster teacher well-being globally.

## Conclusion

This study explored the relationships between teacher job embeddedness, collective efficacy, work meaningfulness, and psychological well-being among Chinese teachers. The findings reveal that both job embeddedness and collective efficacy have direct positive effects on psychological well-being, while work meaningfulness plays a pivotal mediating role in these relationships. The integration of quantitative analysis with qualitative insights through digital ethnography offered a comprehensive understanding of the dynamics underlying teacher well-being. By capturing both statistical correlations and the nuanced lived experiences of Chinese teachers, the study underscores the importance of fostering a supportive school community and strong administrative leadership in promoting teacher well-being. This mixed-methods approach has illuminated how structural and emotional elements within the school environment converge to shape teacher well-being, offering both theoretical and practical insights.

### Implications

The findings of this study hold substantial implications for both theory and practice, shedding new light on the intricate dynamics of teacher well-being, particularly within the Chinese educational system. By integrating the concepts of job embeddedness, collective efficacy, and work meaningfulness, this research not only highlights the direct impact of these factors on teacher well-being but also reveals the nuanced mediating role that work meaningfulness plays in these relationships. This study significantly enriches the existing literature by filling gaps in the understanding of how these constructs interact to influence psychological well-being in specific cultural contexts like China.

From a theoretical perspective, this research advances the discourse on teacher well-being by addressing areas that have received limited attention in previous studies. While the individual effects of job embeddedness and collective efficacy on well-being have been explored, this study offers new insights into how these factors converge, particularly through the mediating influence of work meaningfulness. The findings underscore the central role of collective efficacy in promoting well-being but introduce the critical mediating role of work meaningfulness as a mechanism through which job embeddedness and collective efficacy shape teachers’ psychological well-being. This suggests that future research should delve deeper into mediating variables within models of teacher well-being, as this approach can capture the complex interplay between professional and personal factors more accurately.

Furthermore, this research extends the application of self-determination theory (Deci and Ryan, 2000) by exploring how its core elements—autonomy, competence, and relatedness—are affected by cultural factors, such as collectivism and hierarchical structures within school systems. In a collectivist context like China, the emphasis on relatedness plays a particularly vital role in strengthening both job embeddedness and collective efficacy, which in turn enhances work meaningfulness. These findings suggest that while self-determination theory’s principles are applicable across cultures, the relative importance of its components may shift depending on cultural values. This contributes to cross-cultural psychology by highlighting the need for culturally nuanced applications of well-established theories.

The practical implications for school leaders and policymakers are equally important. Strengthening job embeddedness emerges as a key strategy for enhancing teacher retention and well-being. Schools can prioritize initiatives that build stronger connections among staff, not only in professional terms but also by considering personal and community ties that contribute to overall life satisfaction. For example, mentoring programs, peer collaboration opportunities, and encouraging engagement in community activities can reinforce both on- and off-the-job embeddedness, leading to a more satisfied and stable teaching workforce.

In addition, the study emphasizes the importance of fostering collective efficacy as a way to improve both work meaningfulness and teacher well-being. School leaders can promote collective efficacy by establishing collaborative and inclusive work environments. This can be achieved through practical measures such as forming professional learning communities (PLCs), facilitating team-based lesson planning, and creating structures that encourage peer support and shared decision-making. Such collaborative environments foster a shared sense of purpose and collective responsibility for student success, directly enhancing teachers’ sense of efficacy and contributing to overall well-being.

Moreover, the mediating role of work meaningfulness points to the necessity for schools to ensure that teachers find purpose in their roles. Clear communication of the school’s broader mission and vision, along with alignment of individual goals to this vision, can create pathways for teachers to reflect on the significance of their work. Recognizing and celebrating both individual and collective accomplishments can further enhance the sense of meaning teachers derive from their work, thereby sustaining their commitment and motivation.

The qualitative findings from this study also underscore the importance of addressing work-life balance, a critical concern in the teaching profession. Teachers overwhelmed by administrative burdens and excessive workloads often struggle to maintain this balance, leading to burnout and reduced job satisfaction. Schools can alleviate this by implementing policies that ensure manageable workloads, reducing unnecessary administrative tasks, and offering flexible scheduling where possible. A focus on work-life balance is essential for sustaining long-term job embeddedness and preventing teacher attrition.

At the policy level, this study highlights the need for a more holistic approach to teacher support, with comprehensive well-being programs that integrate job embeddedness, collective efficacy, and work meaningfulness. Such programs should move beyond professional development to address the socio-emotional aspects of teaching, promoting collaboration, mental health support, and positive school climates. Importantly, the study also highlights the cultural dimensions of teacher well-being, suggesting that policies and initiatives be tailored to reflect culturally specific values, such as collectivism and social harmony. In collectivist contexts, fostering a sense of community and shared purpose is likely to have a greater impact on teacher well-being than approaches that emphasize individual achievement.

In terms of its broader contributions to the field, this study deepens the understanding of the complex factors that influence teacher well-being, particularly in the under-explored context of Chinese education. By adopting a mixed-methods approach that integrates both quantitative and qualitative data, the research provides a multi-dimensional perspective on how job embeddedness, collective efficacy, and work meaningfulness interact to shape teacher well-being. The inclusion of digital ethnography as a methodological tool demonstrates the value of exploring online communities as a source of rich qualitative data, offering a model for future studies that seek to capture the nuanced experiences of teachers in various cultural and organizational settings.

### Limitations

The present study offers valuable insights, but some limitations warrant consideration. First, the study employed a self-report design, which is susceptible to common method bias. This reliance on self-reported data for all variables increases the possibility that the findings may be inflated due to participants’ tendency to provide answers that appear consistent across the survey. Future research could benefit from incorporating multiple data sources, such as teacher observations or student perceptions, to triangulate the findings and strengthen the overall validity of the results.

Second, the study focused on a sample of Chinese teachers. While the findings provide valuable context within the Chinese educational system, where collectivism and social connectedness are highly valued (Bond and Smith, 1996), it is important to acknowledge potential cultural limitations. The relationships between job embeddedness, collective efficacy, work meaningfulness, and well-being may differ in educational systems that emphasize individualism or prioritize other cultural values. Future research can explore the generalizability of these relationships across diverse cultural contexts to gain a more comprehensive understanding of these factors influencing teacher well-being on a global scale.

Additionally, the qualitative phase of the study, while providing rich and nuanced insights, also presents certain limitations. The digital ethnography relied on interactions within two specific online communities, which may not fully represent the broader population of Chinese teachers. The nature of online interactions can sometimes limit the depth of responses due to the absence of face-to-face communication cues. Furthermore, the voluntary participation in online discussions might have attracted teachers who are more comfortable with digital communication, potentially biasing the sample.

Future qualitative research could benefit from including a wider range of online platforms and incorporating in-depth interviews or focus groups conducted in person to capture a broader spectrum of teacher experiences. Despite these limitations, the present study offers a significant contribution to the existing research on teacher well-being. By investigating the interplay between job embeddedness, collective efficacy, work meaningfulness, and well-being in a sample of Chinese teachers, and incorporating a qualitative phase through digital ethnography, the study provides deep insights that can inform practices aimed at supporting teacher well-being within the Chinese educational system. This comprehensive approach paves the way for future research to explore these relationships in broader cultural contexts, thereby enriching the global understanding of factors that promote teacher well-being.

## Data Availability

The raw data supporting the conclusions of this article will be made available by the authors, without undue reservation.
